# In vivo enrichment of busulfan-resistant germ cells for efficient production of transgenic avian models

**DOI:** 10.1038/s41598-021-88706-6

**Published:** 2021-04-28

**Authors:** Young Min Kim, Kyung Je Park, Jin Se Park, Kyung Min Jung, Jae Yong Han

**Affiliations:** 1 Department of Agricultural Biotechnology and Research Institute of Agriculture and Life Sciences, College of Agriculture and Life Sciences, Seoul National University, 1 Gwanak-ro, Gwanak-gu, Seoul, 08826 Korea; 2 Institute for Biomedical Sciences, Shinshu University, Minamiminowa, Nagano, 399-4598 Japan

**Keywords:** Biotechnology, Animal biotechnology, Cloning, Genetic engineering

## Abstract

Most transgenic animals are generated using a genome-modified stem cell system and genome modification directly in embryos. Although this system is well-established in the development of transgenic animals, donor cell-derived transgenic animal production is inefficient in some cases. Especially in avian models such as chickens, the efficiency of transgenic animal production through primordial germ cells (PGCs) is highly variable compared with embryonic manipulation of mammalian species. Because germ cell and germline-competent stem cell-mediated systems that contain the transgene are enriched only at the upstream level during cell cultivation, the efficiency of transgenic animal production is unreliable. Therefore, we developed an in vivo selection model to enhance the efficiency of transgenic chicken production using microsomal glutathione-S-transferase II (*MGSTII*)-overexpressing PGCs that are resistant to the alkylating agent busulfan, which induces germ cell-specific cytotoxicity. Under in vitro conditions, *MGSTII-tg* PGCs were resistant to 1 μM busulfan, which was highly toxic to wild-type PGCs. In germline chimeric roosters, transgene-expressing germ cells were dominantly colonized in the recipient testes after busulfan exposure compared with non-treated germline chimera. In validation of germline transmission, donor PGC-derived progeny production efficiency was 94.68%, and the transgene production rate of heterozygous transgenic chickens was significantly increased in chickens that received 40 mg/kg busulfan (80.33–95.23%) compared with that of non-treated germline chimeras (51.18%). This system is expected to significantly improve the efficiency of generating transgenic chickens and other animal species by increasing the distribution of donor cells in adult testes.

## Introduction

Transgenic animals are a powerful tool that allow functional investigations of specific genes, production of recombinant proteins, tissue implantation models in biomedical research, and comparative studies between animal models and humans. To generate transgenic models that permanently express a transgene, two major methods have been employed. Firstly, microinjection of exogenous DNA into the nuclei of embryos is used to produce genetically modified animals, including mice, rabbits, sheep, and pigs^1–4^. Recently, these techniques have been combined with genome editing systems, including zinc finger nucleases (ZFN), transcription activator-like effector nuclease (TALEN), and the clustered regulatory interspaced short palindromic repeats (CRISPR) system to produce model animals of various genotypes, including tissue-specific gene knockout and transgene expression^5–8^. Furthermore, many studies using the CRISPR/Cas9 system have demonstrated that production of genetically modified animals is quite high using this system^8–10^. On the other hand, injection of genetically modified pluripotent stem cells, including embryonic stem cells (ESCs) and induced pluripotent stem cells (iPSCs), into the cavity of blastocysts has also been utilized. This gene transfer system holds advantages over in vitro selection strategies, as it allows development of homogenous transgene‐expressing cell lines and precise gene targeting for efficient production of transgenic animal models^11,12^. Despite its advantages, the production rate of transgenic animals from chimera developed using genetically modified stem cells are not consistent or guaranteed, even after drug screening^13,14^.

Unlike mammals, in avian systems such as the chicken, animals can be produced using primordial germ cells (PGCs), the progenitor cells of both sperm and ovum, and PGCs can be genetically modified for production of transgenic progenies^15^. Recent progress in long-term culture of chicken PGCs and genome modification techniques have allowed the development of numerous transgenic and genome-edited chickens^16–23^. At this point, the chicken is considered to be a reliable biological model representing avian species. As shown in prior studies, the chicken PGC-mediated transgenic production system can also increase production efficiency through drug selection during in vitro cultivation. Nevertheless, the production of PGC-derived genetically modified chickens is not consistent, and production efficiency is not guaranteed, similar to the stem cell-mediated system used for mammalian species. Some prior reports demonstrated that the efficiency of producing transgenic progeny derived from donor cells was consistently higher than 50%^17,24^. Theoretically, the efficiency of heterozygotic transgenic production is 50% at maximum because the transgene of donor PGCs is integrated into haploid germ cells. However, in most cases, germline transmission efficiency and transgenic progeny production rates are varied and low^18–20,22,23^.

The efficiencies of transgenic animal production using the mouse stem cell-mediated system and chicken PGC-mediated system are limited, as it is only possible to increase homogenous transgene‐expressing cell lines at the upstream stage of donor cell cultivation. These systems are inherently limited, as the proportion of transgene-expressing donor cells cannot be regulated, and transgene insertion is irreversible after transfer to recipient embryos. To overcome this limitation, several means to sterilize the recipients’ endogenous germ cells to increase the proportion of donor cells have been used in chickens.

Sterilization of endogenous germ cells is often used for germline chimera production. Sterilization techniques include gamma ray irradiation, X-ray irradiation, and chemical methods to eliminate endogenous germ cells^25–29^. In particular, the alkylating agent busulfan induces relatively high germ cell-specific cytotoxicity, including both PGCs and spermatogonial cells in adult testes^30,31^. Busulfan is also considered to be a very effective antispermatogonial agent in avian testes^32,33^, and allows for highly efficient germline chimera production when used to treat unhatched chicken embryos^27,34^. However, despite the finding that busulfan causes testicular germ cell apoptosis, a small number of spermatogonial stem cells (SSCs) survive and restore the germ cell population^35^. This suggests that normal spermatogenesis can be maintained after endogenous germ cells are eliminated by busulfan.

In a previous report, Harkey et al. demonstrated that overexpression of glutathione-S-transferase (GST) genes (*GSTA1*, *GSTP1*, and *MGSTII*) in HEK cells for hematopoietic gene therapy is significantly increased using the busulfan resistance system^36^. Specifically, the *MGSTII* gene conferred a reproducible twofold selective advantage under busulfan exposure conditions, suggesting that this approach could be used for hematopoietic gene therapy^36^. The GST enzymes catalyze the conjugation of a variety of small molecules, including alkylating agents such as busulfan, with glutathione (GSH), targeting these molecules for export^36,37^. This property enables detoxification of alkyl agents in GST-expressing cells. In the present study, we developed an in vivo selection system for busulfan-resistant transgene (*MGSTII*)-containing chicken germ cells in adult testes as well as in vitro selection. The selection of transgenic germ cells in the adult stage of germline chimera is considered to be highly advantageous for the reproduction of donor-derived progeny. To test the reliability of this system, we produced a germline chimera containing a busulfan resistance gene, and subsequently assessed the efficiency of transgenic progeny production using busulfan treatment.

## Results

### Establishment of a MGSTII expressing chicken PGC line with in vitro resistance to busulfan.

**Figure 1:**
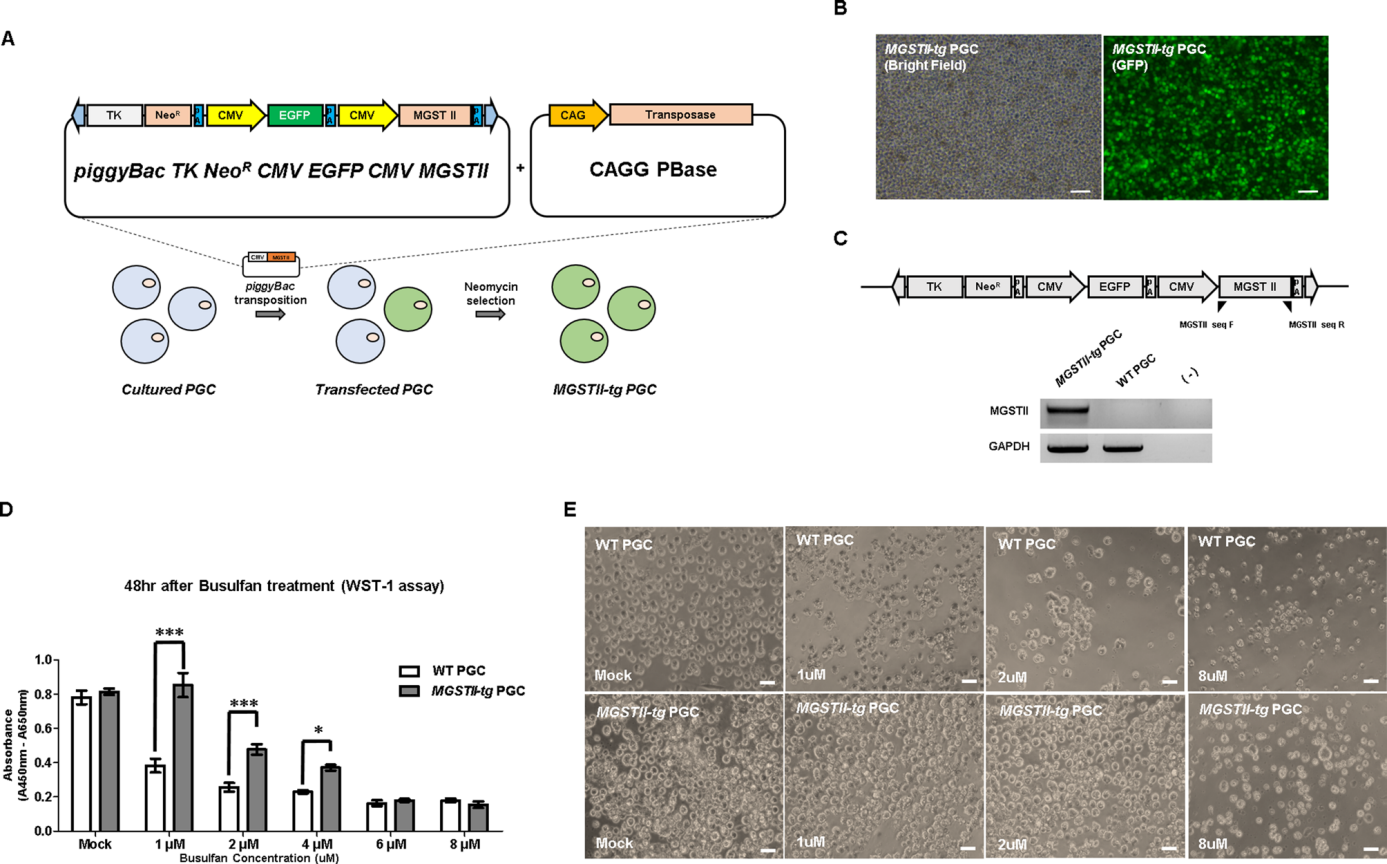
Establishment of the *piggyBac CMV EGFP CMV MGSTII* PGC cell line and validation of busulfan effect on MGSTII-expressing PGCs. (**A**) Schematic illustration of the *piggyBac TK Neo*^*R*^* CMV EGFP CMV MGSTII* vector with transposase and introduction into PGCs. Transgenic PGCs were selected with G418 for 30 days. (**B**) Establishment of the *piggyBac TK Neo*^*R*^* CMV EGFP CMV MGSTII* transgenic PGC cell line (*MGSTII-tg* PGCs) after selection. Scale bars, 200 μm. (**C**) Genomic DNA PCR analysis of *MGSTII-tg* PGCs using *MGSTII-* and *GAPDH-*specific primers. Wild-type (WT) PGCs treated with distilled water (−) were used as control. (**D–E**) Dose-dependent effect of busulfan on *MGSTII-tg* and WT PGCs. (**D**) WST1 assay of PGCs after 48 h of dose-dependent busulfan treatment (mean ± SD; n = 3). * *P* < 0.05, **P < 0.001. (**E**) Morphology of *MGSTII-tg* and WT PGCs in the presence of multiple busulfan dosages (0, 1, 2, and 8 μM). Vehicle control was treated with DMSO only. Scale bars, 50 μm.

To establish *MGSTII*-expressing chicken PGCs, we first constructed a *piggyBac* transposon vector designed for CMV-driven expression of enhanced green fluorescent protein (*EGFP*) and chickenized human *MGSTII*. Subsequently, cultured PGCs were transfected with *piggyBac TK Neo*^*R*^* CMV GFP CMV MGSTII* and transposase (CAGG-PBase) plasmids (Fig. 1A). Transfected PGCs (*MGSTII-tg* PGCs) were selected with G418 (300 μg/mL) culture medium for 1 month (Fig. 1B). *MGSTII-tg* PGCs exhibited robust GFP fluorescence and specifically expressed the *MGSTII* gene compared with WT PGCs (Fig. 1C). To analyze busulfan resistance in *MGSTII-tg* PGCs, we analyzed the time- and dose-dependent effects of busulfan treatment on cell viability. The effect of busulfan on WT and *MGSTII-tg* PGC proliferation was examined using a WST-1 assay after 48 h exposure to varying concentrations of busulfan. In cells treated with 1 μM, 2 μM, and 4 μM busulfan, the proliferation rate of *MGSTII-tg* PGCs was significantly higher than that of WT PGCs (Fig. 1D). In cultured PGCs, 1 μM and 2 μM busulfan exposure decreased cell numbers in WT PGCs relative to *MGSII-tg* PGCs, but 8 μM busulfan decreased the number of PGCs in both genotypes (Fig. 1E). These results suggested that *MGSTII-tg* PGCs had increased resistance to busulfan, and therefore that *MGSTII* overexpression conferred resistance to busulfan.

### Effect of busulfan on migrating PGCs

**Figure 2:**
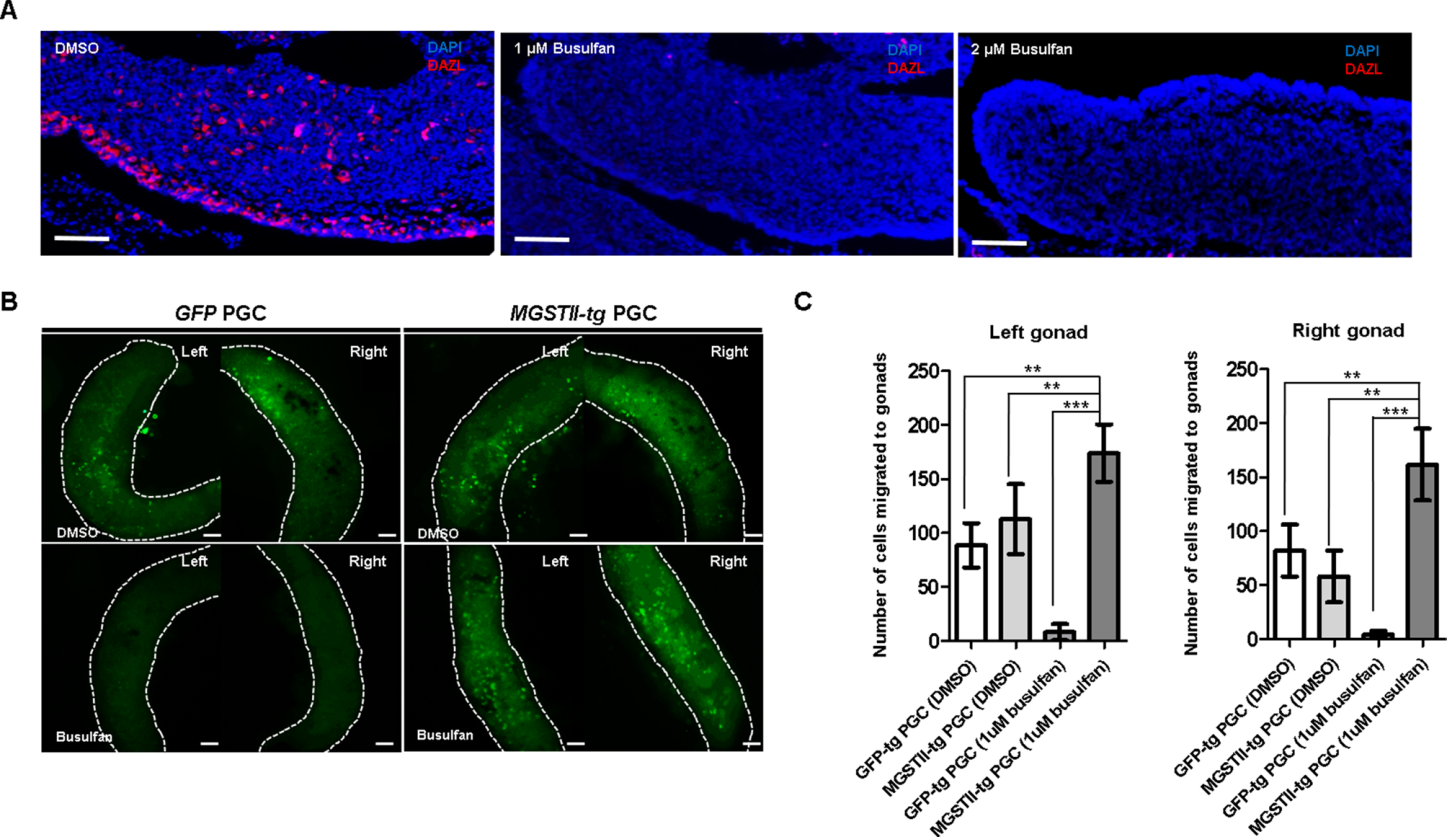
Effect of busulfan in embryonic gonads after *MGSTII-tg* PGC transplantation. (**A**) In vivo effect of chicken PGCs under busulfan treatment. Endogenous PGCs were detected by DAZL immunostaining in HH28 embryonic gonads with or without busulfan treatment. Scale bars, 100 μm. (**B**) Migration assay of *MGSTII-tg* and *GFP-tg* PGCs treated with 1 μM busulfan. Approximately 1,000 *GFP-tg* or *MGSTII-tg* PGCs were injected into the dorsal aortas of chicken embryos at HH 13–16. Fluorescent cells were observed and counted in recipient embryonic gonads at HH 28. Scale bars, 100 μm. (**C**) Number of PGCs migrated to the gonads of HH 28 embryos injected (i.v.) with 1,000 PGCs at HH 13–16 (mean ± SD; n = 3 for each group). *P < 0.05, **P < 0.01, ***P < 0.001.

In subsequent studies, we examined the effect of busulfan on migrating PGCs in the blood stream and to the genital ridge. Avian PGCs circulate through embryonic blood vessels during HH 13–16 and migrate to the gonads by HH 28. Thus, to assess the effect of busulfan on embryonic PGCs, we injected 1 μM and 2 μM of busulfan into embryonic blood vessels at stage HH 13–16. At embryonic day 5.5 (HH 28), whole gonads were collected from control and busulfan-treated embryos, and DAZL immunostaining was conducted to identify germ cell distribution. In control gonads (animals injected with DMSO vehicle control), DAZL^+^ PGCs were dispersed in the entire gonad, but DAZL^+^ PGCs were completely eliminated in gonads of the 1 μM and 2 μM busulfan-treated groups (Fig. 2A).

To assess busulfan resistance in embryonic stages, we further conducted an in vivo migration assay using *MGSTII-tg* PGCs exposed to busulfan. Approximately 1,000 *GFP-tg* PGCs^17^ or *MGSTII-tg* PGCs were injected into the bloodstream of recipient HH 13–16 embryos with or without 1 μM busulfan. In untreated PGCs, the number of PGCs was similar in whole embryonic gonads at HH 28 in recipient embryos injected with *GFP-tg* PGCs and embryos injected with *MGSTII-tg* PGCs (Fig. 2B). However, in the busulfan-treated embryos, the number of migrated PGCs was dramatically decreased in embryos injected with *GFP-tg* PGCs relative to embryos injected with *MGSTII-tg* PGCs (Fig. 2B). The mean number of migrated gonadal PGCs in the left gonads of embryos injected with 1 μM busulfan-treated *MGSTII -tg* PGCs (174 ± 24.45) was significantly higher than not only in embryos injected with 1 μM busulfan-treated *GFP-tg* PGCs (9 ± 7.54), but also in embryos injected with untreated *GFP-tg* PGCs (88 ± 20.59) and untreated *MGSTII-tg* PGCs (112.67 ± 32.47). Similarly, the mean number of migrated cells in the right gonads of embryos injected with busulfan-treated *MGSTII-tg* PGCs (161 ± 33.08) was also significantly higher than in other groups (82 ± 24.06 in untreated *GFP-tg* PGCs, 58 ± 24.02 in untreated *MGSTII-tg* PGCs, and 4.3 ± 3.21 in busulfan-treated *GFP-tg* PGCs) (Fig. 2C). These results suggested that *MGSTII-tg* PGCs were resistant to busulfan in vivo, as demonstrated by in vivo migration activity. We also examined the survivability and hatchability of recipients after transfer of PGCs with busulfan treatment into embryonic blood vessels (at HH 13–16) (Supplementary Table 1). The survivability (at E6) and hatchability of recipients were 50% lower in the busulfan only-treated group that in the untreated control group. The survivability (at E6) and hatchability of recipients were not lower in the busulfan-treated/untreated groups injected with transgenic PGCs (*GFP-tg* PGCs and *MGSTII-tg* PGCs) than in the busulfan only-treated group (Supplementary Table 1). These results suggest that busulfan does not directly affect the development or hatching of recipient embryos.

**Table 1:** Efficiency of germ-line transmission and transgenic chick production with *MGSTII-tg* donor PGCs.

Germline chimera ID	No. of incubated eggs	No. of hatched chicks (%)	No. of donor germ cell–derived chicks (%)^†^	No. of transgenic chicks (%)^‡^
M 0398	84	79 (94.05)	63 (79.74)	33 (52.38)
M 0412	58	41 (70.69)	31 (75.61)	14 (45.16)

^†^The phenotype of offspring derived from donor PGCs of WL chickens (*I/I*).

^‡^The percentage of donor germ cell–derived chicks that expressed EGFP.

### Production of MGSTII transgenic chickens and detection of genomic integration site

**Figure 3:**
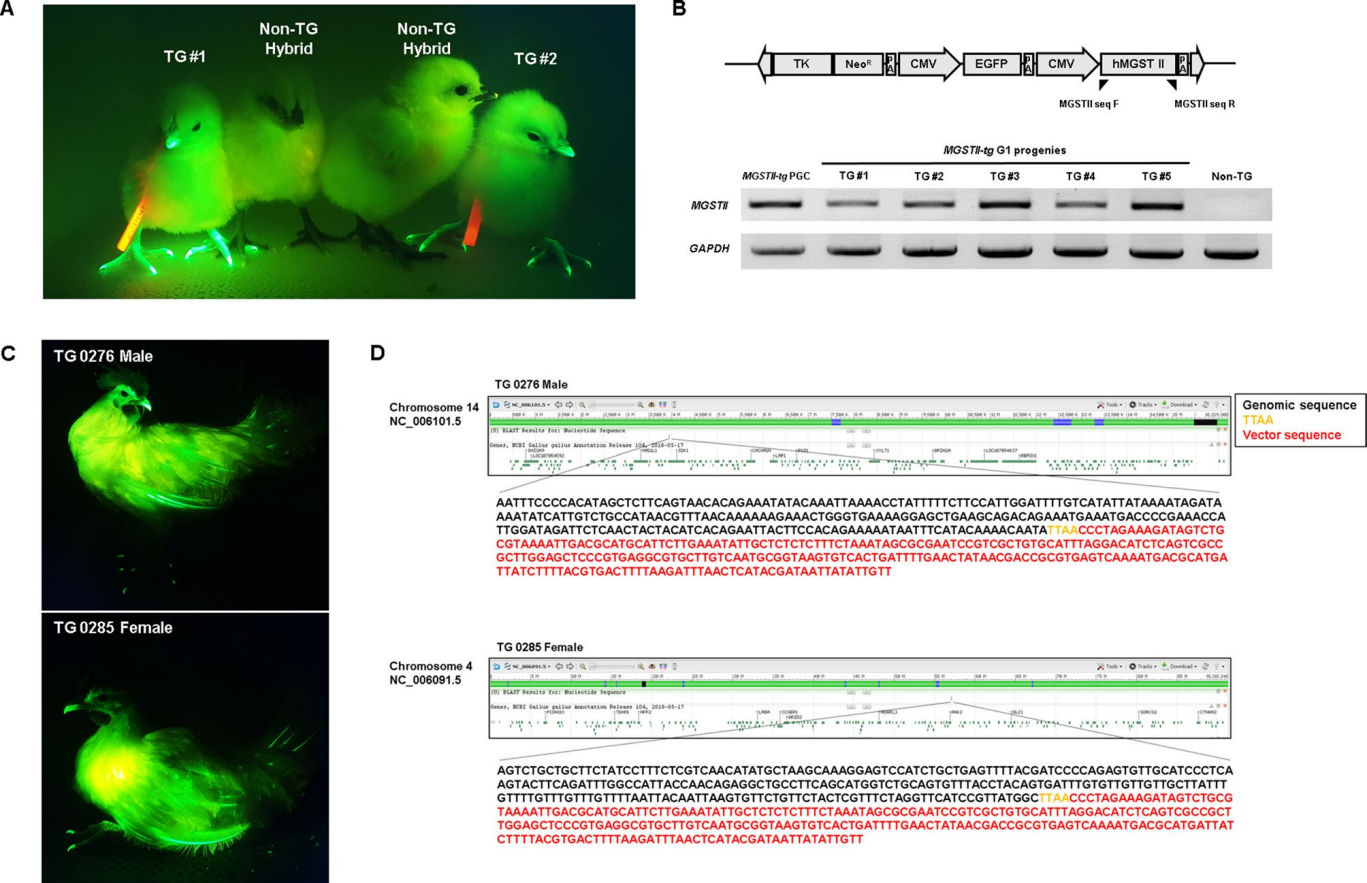
GFP expression in of *MGSTII-tg* chicks and identification of transgene insertion sites. (**A**) Donor *MGSTII-tg* PGCs-derived chicks. GFP-expressing chicks were distinguishable from non-transgenic hybrids using a fluorescent excitation lamp fitted with the appropriate filters. (**B**) Screening of *MGSTII-tg* chicks via genomic DNA PCR analysis. Genomic DNA PCR analysis of transgenic G_1_ chicks using *MGSTII*- and *GAPDH-*specific primers. *MGSTII-tg* PGCs and non-tg genomic DNA samples were used as positive and negative controls, respectively. (**C**) Detection of GFP expression in adult TG chickens (20 weeks) in both male (TG 0276) and female (TG 0285) animals using a fluorescent excitation lamp fitted with the appropriate filters. (**D**) Identification of the transgene integration site in transgenic chickens. The transgene was integrated into the intergenic region of chromosome 14 in TG 0276, and into the intergenic region of chromosome 4 in TG 0285. Black color nucleotides indicate genomic DNA sequences, yellow color nucleotides indicate transposon recognition sequences (TTAA), and red color nucleotides indicate the 5′ of *piggyBac* vector sequences.

To produce h*MGSTII*-expressing transgenic chickens, cultured *MGSTII-tg* PGCs were injected into HH 13–16 Korean Ogye (KO) chicken embryos. A total of 39 embryos were injected with *MGSTII-tg* PGCs and 21 hatched (hatchability was 53.84%) (Supplementary Table 2). The donor cells (*MGSTII-tg* PGCs) were male PGCs from White Leghorn (WL) chickens; therefore, only male KO recipients were selected as putative germline chimeras by genomic DNA sexing PCR (data not shown). Eight progenies were determined to be male (0396, 0398, 0399, 0411, 0412, 0415, 0416, and 0418). After development for about 5 months, five founder males (0398, 0399, 0411, 0412, and 0416) reached sexual maturity. Among these, two germline chimeric chickens (0398 and 0412: hereafter, a “M” is placed in front of the animal number to designate founder germline chimeras e.g., “M0398” and “M0412”) were randomly selected and testcrossed with wild-type WL hens (Supplementary Table 2). The efficiencies of donor *MGSTII-tg* PGC-derived chicks from two germline chimeras (M0398 and M0412) were 79.74% and 75.61%, respectively (Table 1). Among the donor-derived progeny, transgenic chicks were validated through GFP expression, which was absent in non-transgenic chicks (Fig. 3A). Analyses of GFP expression and transgene genomic PCR were performed, identifying that approximately half of donor germ cell-derived chicks were transgenic (33 of 63 chicks, 52.38% from M0398, and 14 of 31 chicks, 45.16% from M0412) (Fig. 3B, Table 1). In G1 transgenic chickens, GFP expression was measured in one transgenic male (TG0276) and one transgenic female (TG0235), and robust GFP expression was detected until sexual maturation (Fig. 3C). In subsequent analyses, the genomic transgene integration sites were identified by genome walking analysis. The *piggyBac TK Neo*^*R*^* CMV GFP CMV MGSTII* transgene was integrated into the intergenic region of chromosome 14 in TG0276 and into the intergenic region of chromosome 4 in TG0285 (Fig. 3D). Both loci had conserved TTAA sequences, in which the *piggyBac* transposon could be inserted by transposase.

### Enhanced transgenic chicken production rate after busulfan treatment

**Figure 4:**
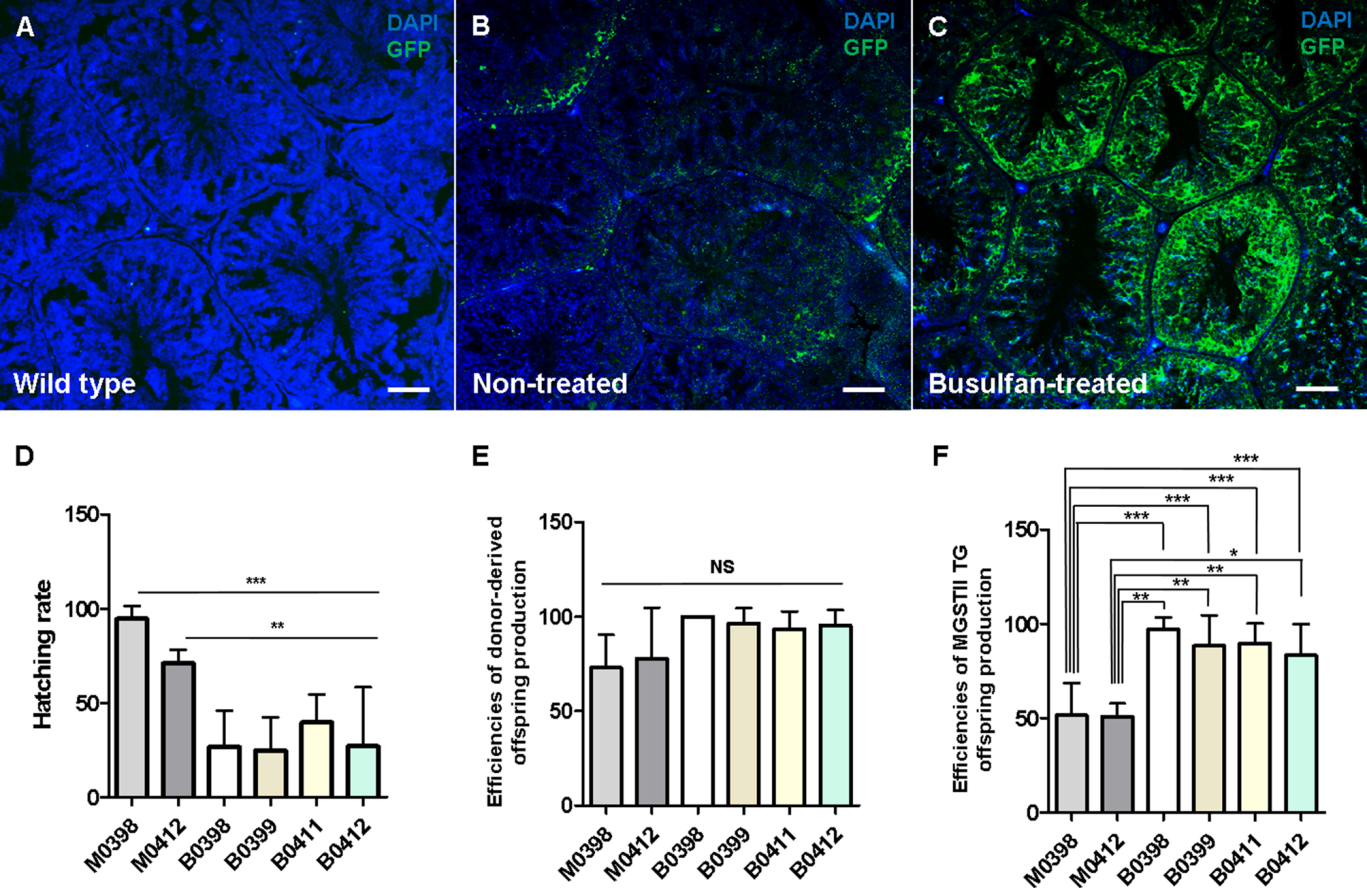
Improved transgenic chicken production rate after busulfan injection. (**A–C**) Cross-sections of testes from adult germline chimeras, and detection of GFP-expressing donor germ cells in adult germline chimeric testes with or without busulfan treatment. (**A**) WT rooster testes were used as a negative control. GFP expression was not observed. (**B**) Testes of germline chimeric roosters without busulfan treatment. GFP^+^ cells were sparsely distributed adjacent to the basement membranes of seminiferous tubules. (**C**) Testes from germ chimeric roosters 2 weeks after busulfan treatment. GFP^+^ cells were present throughout the seminiferous tubules. DAPI was used to stain nuclei in each panel. Scale bars, 200 μm (upper panel) and 50 μm (lower and smaller panel). (**D–F**) Analysis of progeny production rates of germline chimera for 8 weeks. (**D**) Hatching rate of G_1_ offspring from germline chimeras with or without busulfan treatment. (**E**) Efficiencies of donor-derived offspring production with or without busulfan treatment. (**F**) Efficiencies of *MGSTII-tg* offspring (G_1_) production with or without busulfan treatment. Data are presented as means ± SD from each independent group (progeny production of each week) for 8 weeks. *P < 0.05, **P < 0.01, ***P < 0.001.

After evaluating the donor-derived offspring and transgenic chicken production rates in untreated animals, the same germline chimeras (M0398 and M0412) and two additional germline chimeras (M0399 and M0411) received a single intraperitoneal injection of 40 mg/kg busulfan. Busulfan-treated germline chimeras were designated by placing a “B” in front of the animal number, for example B0398, B0399, B0411, and B0412. To confirm the distribution of GFP^+^ germ cells in testes, 10 µm paraffin sections from germline chimera WT and busulfan non-treated control testes were examined under a fluorescent microscope after immunostaining for GFP as a marker for transplanted transgenic germ cells (Fig. 4A–C). In WT testes, GFP^+^ cells were not observed (Fig. 4A), but in testes of germline chimera untreated with busulfan (M0416), GFP^+^ germ cells were sparsely dispersed adjacent to the basements of seminiferous tubules (Fig. 4B). Surprisingly, the distribution of GFP^+^ germ cells in testes of busulfan-treated germline chimera was significantly increased compared with non-treated controls and WT testes (Fig. 4C). To determine the increase in transgenic offspring production efficiency, busulfan-treated germline chimeras were testcrossed by insemination of female WL chickens. Unexpectedly, all busulfan-treated germline chimeras exhibited a decreased hatch rate (35.59–45.31%) compared with the non-treated control group (70.69–94.05%) even in the same germline chimeras used in the busulfan non-treated group (B0398, B0412) (Tables 1, 2, Fig. 4D). Despite the higher efficiency of donor-derived offspring production in the busulfan-treated group relative to control (94.68% vs. 78.33%, respectively), this relationship was not statistically significant (Tables 1, 2, Fig. 4E). However, the production rate of transgenic chickens was significantly enhanced in the busulfan-treated group (80.95%) relative to the non-treated group (45.16%) (Tables 1, 2, Fig. 4F). In particular, transgenic chicken production efficiency was significantly improved by busulfan treatment even in the same germline chimeric roosters. The transgenic chicken production efficiency was significantly improved by busulfan treatment, from 52.38 to 95.23% in M0398 and 45.16% to 80.95% in M0412.

**Table 2:** Efficiency of germ-line transmission and transgenic chick production with *MGSTII-tg* donor PGCs after busulfan treatment.

Germline chimera ID	No. of incubated eggs	No. of hatched chicks (%)	No. of donor germ cell–derived chicks (%)^†^	No. of transgenic chicks (%)^‡^
B 0398	59	21 (35.59)	21 (100)	20 (95.23)
B 0399	55	22 (40.0)	20 (90.90)	16 (80.0)
B 0411	64	29 (45.31)	27 (93.10)	24 (88.8)
B 0412	57	22 (38.59)	21 (95.45)	17 (80.95)

^†^The phenotype of offspring derived from donor PGCs of WL chickens (*I/I*).

^‡^The percentage of donor germ cell–derived chicks that expressed EGFP.

## Discussion

The production of transgenic cells and organisms provides a platform to assess gene functions, improve human health, enhance production of useful materials, protect against environmental threats, and control disease in livestock^1^. In this regard, the conventional means for transgenic animal production, involving introduction of foreign genomic materials into fertile eggs, and newer genome editing technologies such as CRISPR/Cas9 have been successfully utilized. In mammalian systems, genome editing systems are highly efficient, with the desired genotype reaching almost 100%^8–10^. If only small gene deletions are required, further technology development might not be necessary.

However, production of transgenic animals through transgene overexpression and targeted gene insertion in germline-competent stem cells is still required in some contexts such as for gene homologous recombination and precise targeted gene insertion^38^. Thus far, cell-mediated systems do not efficiently and reliably produce transgenic animals. At the initial stage, the frequency of stable integration into the genomes of host cells is very low. Therefore, transgene cassettes that include resistance genes to dominant selection markers have been used to increase efficiency; for example, *neomycin phosphotransferase II* against the neomycin derivative G418, *puromycin N-acetyltransferase* against puromycin, and *hygromycin B phosphotransferase* against hygromycin B^39^. This strategy can induce homogenization of transgene-integrated cells and supply the appropriate cell population for transgenesis. Despite drug selection for transgenic cell lines, the efficiency of desired transgenic littermate production is not enhanced, as the process is irreversible after transfer to recipient embryos. To overcome this limitation, a method to increase the proportion of donor cells in vivo is required.

A recent study demonstrated that transgenic chickens expressing a fluorescent protein (mCherry or GFP) and avian leukosis virus (ALV)-resistant genome-edited chickens are effectively produced by transplanting PGCs into sterilized recipient testes^40,41^. Despite the relatively high production efficiency (more than 60% in some cases), this method requires intramagnal insemination, which may limit mass production of transgenic/genome-edited progenies due to technical difficulties. Another research group reported a germ cell sterilized model through targeted *DDX4* gene disruption^20^, which enables production of donor-derived offspring with high efficiency using *DDX4*-knockout embryos as recipients^42^. More recently, another germ cell sterilized recipient model was generated by conditional knockout of the *DAZL* gene^43^. These methods can effectively increase the proportion of donor cells in the recipient and therefore be used to effectively conserve rare breeds and produce transgenic animals. However, germ cell sterilized model systems must be established separately to produce the proposed animal, which limits their wide application.

In the present study, we demonstrated that transgenic chicken production efficiency was increased in vivo by use of a busulfan resistance cassette, as busulfan is specifically toxic to endogenous germline cells in chimeric animals. Busulfan, an alkylating agent with antispermatogonial activity, is highly cytotoxic to germ cells specifically, including PGCs and SSCs in mouse, rat, and avian testes^30,31,33^. Busulfan treatment of chicken embryos also increased germline chimera production^27,34^. Busulfan induces germ cell damage and germ cell apoptosis mediated by loss of the c-kit/SCF pathway, a mechanism that is conserved in multiple germ line cell types, including PGCs and spermatogonia^44^. It was therefore expected that high-efficiency transgenic animal production would be possible simply by developing busulfan-resistant germ cells expressing the transgene of interest. Previous studies^36^ suggested that overexpression of human GST, *MGSTII*, confers resistance to busulfan in HEK293 cells. In this regard, we overexpressed *hMGSTII* in chicken PGCs using the *piggyBac* transposon, and confirmed significant resistance to 1 μM, 2 μM, and 4 μM busulfan relative to WT PGCs. Interestingly, busulfan is cytotoxic not only to PGCs in early embryos^29,34^, but also to circulating PGCs in blood vessels after exogenous PGC transplantation. Moreover, the number of PGCs migrated to the recipients’ gonads (both left and right) was significantly increased by treatment of *MGSTII-tg* PGCs with busulfan prior to transplantation, and was increased relative to both vehicle- and busulfan-treated *GFP-tg* PGCs. We also evaluated the harmful effect of busulfan treatment on developing embryos under several conditions. Although survivability was lower in all experimental groups than in the untreated control group, the hatching rate did not significantly differ between the busulfan-treated (busulfan only and transgenic PGCs with busulfan) and untreated (transgenic PGCs without busulfan) groups. Therefore, egg shell windowing and cell/busulfan injection are considered to cause stress, not busulfan itself. These results indicate that use of the *MGSTII* transgene to confer busulfan resistance improved the migration efficiency of transplanted PGCs, as busulfan decreases endogenous PGCs without eliciting other side effects on embryo development and hatching.

In our previous reports, germline transmission efficiency was 90.4–98.9%, and over half of the transgenic chicks (52.2%) were heterozygous^17^. In another study, the *2* × *ERE-OVcEGF* transgene was integrated into chicken PGCs using the *piggyBac* transposon, and the average rate of germline transmission was 92.2%, with 47.1% of transgenic animals being heterozygous^24^. However, these high efficiencies are not always consistent in chicken germline chimeras. In two types of chickens produced using a similar system, the efficiency of germline transmission and transgenesis was highly variable and inefficient^19^, and only one transgenic chick were obtained from 518 progeny chicks (0.193%)^16^. Further, this variance in germline transmission efficiency is exacerbated when combined with targeted genome editing technologies. In targeted knockout of chicken *ovalbumin* in chicken PGCs using TALEN, the germline transmission efficiency was 40.84%, and only 8.04% of progeny had disruption of genomic *ovalbumin*^18^. In a study in which the *GFP* gene was knocked in to the chicken sex chromosome (Z chromosome) using TALEN, 17 targeted genome-edited chicks were derived from 372 hatched chicks^20^. Use of the CRISPR/Cas9 system on chicken PGCs resulted in germline transmission efficiency of 3.1%, and 62.5% of donor germ cell-derived progenies were genome modified^22^. These phenomena are due in part to multicopy transgene integration in transgenic models, and non-homogenous selection of genome-edited germ cells during the drug screening process.

In the present study, the germline transmission efficiencies were 79.74% and 75.61% in two different germline chimera (M 0398 and M 0412), and almost half of the progeny exhibited GFP fluorescence and transgene integration (52.38% and 45.16%, respectively). After intraperitoneal injection of a single dose of busulfan emulsion (40 mg/kg) as in previous reports using chicken and quail^33,45^, germline transmission efficiency was increased to 94.68% on average. Further, the production efficiency of transgenic progeny after busulfan treatment improved to 80.95%. Among the chimeras tested, in the 0398 and 0412 germline chimera used in both the busulfan-treated groups and non-treated groups, the transgenic progeny production efficiency was 95.23% and 80.95%, respectively. However, the fertility of germline chimeras after treatment with busulfan was significantly decreased. In control germline chimeras, the fertility of germline chimeras after busulfan treatment was only 38.59%, which was significantly lower than that of chimeras without busulfan treatment (70.68%). One possible explanation is that SSCs have relatively high resistance to busulfan^44^, and from a haploid round spermatid stage are extremely sensitive to alkylating agents such as cyclophosphamide^46^. Therefore, diploid cells, including spermatogonia and spermatocytes, could be resistant to busulfan-induced cytotoxicity, while the transgene-free haploid germ cells such as spermatocytes, spermatids, and sperm of donor cells and endogenous germ cells were depleted. In those stages, even haploid germ cells from donor-derived cells are highly susceptible to busulfan. As a result, the entire population of germ cells decreases, and haploid germ cells containing the *MGSTII* transgene are relatively increased, such that fertilization rate is decreased, but production efficiency of transgenic progeny is increased.

In transgenic animal production, the use of genetically modified stem cells with germline competency, including pluripotent stem cells and germ cells such as PGCs and SSCs, is required not only for transgene overexpression in target cells, but also for targeted gene insertion and homologous recombination. Thus far, strategies to increase production efficiency of transgenic animals have been limited to drug selection during homogenous cell cultivation in vitro. The in vivo selection system developed in the present study using a busulfan resistance gene combined with busulfan treatment demonstrated that donor-derived transgenic production efficiency can be increased even in adult recipients. To confirm the enhancement of in vitro and in vivo donor cell proportions demonstrated in this study, additional experiments using several gene cassettes, including *MGSTII,* and further validation in other model animals are needed. This strategy can be applied in multiple species to improve the production efficiency of transgenic and genome-edited animals.

## Materials and methods

### Experimental animals and animal care

The care and experimental use of chickens was approved by the Institute of Laboratory Animal Resources, Seoul National University. Chickens were maintained according to a standard management program at the University Animal Farm, Seoul National University, Korea. All procedures, including chicken maintenance, reproduction, and sample collection, were governed by standard operating protocols according to a standard management program at the University Animal Farm, Seoul National University and the Animal Genetic Engineering Laboratory at Seoul National University.

### Construction of human MGSTII expression vector

Codons of the human microsomal glutathione-S-transferase II (*MGSTII*) gene were optimised for expression in the hen using the *Gallus gallus* codon database (https://www.kazusa.or.jp/codon). A codon-optimized human *MGSTII* gene were integrated into *piggyBac TK Neo*^*R*^* CMV GFP FRT* backbone vector from our previous study^19^. Briefly, codon-optimized *MGSTII* CDS inserted into backbone vector by using HindIII, NotI restriction enzymes. After that, *CMV MGSTII* cassette was cloned by PCR including XhoI restriction site at the 5′ end and 3′ end. This cloned cassette was integrated into *piggyBac TK Neo*^*R*^* CMV GFP FRT* backbone by using XhoI restriction enzyme and produce final vector *piggyBac TK Neo*^*R*^* CMV GFP CMV MGSTII*.

### Transfection and G418-selection of PGCs

The methods of cultivation, transfection and G418-selection of WL male PGC cells were followed by our previous report^17^. Briefly, male PGCs were cultured on mitotically inactivated mouse fibroblast cells (MEFs) in knockout Dulbecco's Modified Eagle's Medium (KO-DMEM) (Invitrogen, Life Technologies, Carlsbad, CA, USA) supplemented with 20% (v/v) fetal bovine serum (Invitrogen, Life Technologies), 2% (v/v) chicken serum (Sigma-Aldrich, St. Louis, MO, USA), 1 × nucleoside mix (EMD Millipore, Temecula, CA, USA), 2 mM L-glutamine, 1 × nonessential amino acid mix, β-mercaptoethanol, 10 mM sodium pyruvate, 1 × antibiotic antimycotic mix (Invitrogen, Life Technologies), and human basic fibroblast growth factor (10 ng/mL; Koma Biotech, Seoul, Korea). PGCs were cultured at 37℃ in an atmosphere of 5% (v/v) CO_2_ and 60–70% relative humidity. The *piggyBac TK Neo*^*R*^* CMV GFP CMV MGSTII* expression vector and transposase (CAGG-PBase, pCyL43) were cotransfected into the PGC line using Lipofectamine 2000 (Invitrogen, Life Technologies). One day after transfection, G418 (300 mg/mL) was added to culture medium to enable selection of transfected PGCs for 1 month.

### In vitro assay to assess the effect of busulfan resistance on MGSTII-expressing PGCs

WT and *MGSTII-tg* PGCs were incubated at 50,000 cells per well in 96-well tissue culture plates with varying concentrations of busulfan (1, 2, 4, 6, and 8 μM, Sigma-Aldrich) dissolved in dimethyl sulfoxide (DMSO). To assess the busulfan resistance of *MGSTII-tg* PGCs, cells were incubated in the specified concentrations of busulfan for 48 h. After incubation, cell viability was assessed using the WST-1 method (Roche Diagnostic, Basel, Switzerland). Briefly, WST-1 was added (15 μL/well) and cells were incubated for 4 h. The absorbance (A450-A650) of formazan dye produced by metabolically active cells was detected using an Epoch microplate reader (BioTek, Winooski, VT, USA).

### Transplantation of MGSTII-tg PGCs into recipient embryos

To produce germline chimeric chickens through injection of *MGSTII-tg* PGCs into recipient embryos, we made a small hole at the pointed end of each recipient KO egg at Hamburger and Hamilton (HH) stages HH13–16, and microinjected a 2 μL aliquot containing at least 3,000 *MGSTII-tg* PGCs into the dorsal aortas of recipient embryos. Egg holes were sealed with paraffin film, and eggs were incubated with the pointed end down prior to further screening and eventual hatching. To assess the effect of busulfan on *MGSTII-tg* PGCs, approximately 1,000 *GFP-tg* PGCs, as generated in our previous report^17^, or *MGSTII-tg* PGCs were injected with 1 μM busulfan solution at HH13–16. Three days after transplantation, the gonads from a sample of the recipient embryos were collected at HH 28, and the presence of GFP-expressing PGCs in the gonads were imaged by fluorescence microscopy (Nikon) and manually counted.

### Test-cross analysis and validation of transgenic chicken production efficiency

After sexual maturation, putative germline chimeras were testcrossed by mating with WL female chickens to generate transgenic chickens derived from transplanted donor PGCs. Transgenic (TG) chickens were identified using a fluorescent excitation lamp with detection filters (BLS Ltd., Budapest, Hungary), and were confirmed by genomic DNA PCR. To evaluate the donor-derived production efficiency and transgenic chick production efficiency, adult germline chimeras were injected with 40 mg/kg busulfan. Briefly, 40 mg busulfan dissolved in 1 mL *N,N*-dimethyl formamide (Merck, Darmstadt, Germany) was injected intraperitoneally into germline chimeras. Two weeks after busulfan injection, busulfan-treated germline chimeras were testcrossed by mating with wildtype WL female chickens. At least 20 progeny animals from both groups of germline chimeras (busulfan-treated or non-treated) were analyzed for fertilization rate and transgenic chick production efficiency by measuring hatching rates, fluorescence expression, and genotypes for 8 weeks.

### PCR analysis and identification of the transgene integration site

To detect the presence of the integrated transgene and distinguish between the wild-type (WT) and *MGSTII-tg* loci, transgene specific primer sets (MGST F: 5′-CCA CCA TGG CAG GCA ACA GC-3′ and MGST R: 5′-CCG CTC AGA ATT GCC GCC TC-3′) were used. Genomic DNA PCR from WT and *MGSTII-tg* was also performed using *GAPDH* primers (F: 5′-GGT GGT GCT AAG CGT GTT AT-3′; R: 5′-ACC TCT GCC ATC TCT CCA CA-3′) as a control. Each PCR was performed in a total volume of 20 μL containing 100 ng genomic DNA from WT and *MGSTII-tg*, 10 × PCR buffer (BioFACT, Daejeon, Korea), 10 mM dNTPs, 5 pmol of each primer, and 0.5 U Taq polymerase (BioFACT) in the following thermocycling conditions: 5 min at 94 °C, followed by 35 cycles of 30 s at 94 °C, 30 s at 60 °C, 30 s at 72 °C, and, finally, 10 min at 72 °C.

The transgene (*piggyBac CMV GFP CMV MGSTII TK Neo*^*R*^) insertion site was identified using the Genome Walker Kit (Takara, Japan) according to the manufacturer’s protocol and our previous report^24^. Gene-specific primers were designed from the known DNA sequence of the *piggyBac* transgene to move upstream of the gene in genomic DNA. Genomic DNA was digested with four different restriction enzymes (*EcoRV*, *DraI*, *PvuII*, and *SspI*) ligated with adaptors, and PCR was performed using the polymerase mix. PCR products were excised from agarose gel, purified using a Power Gel Extraction Kit (Promega, Madison, WI, USA), and subsequently cloned into the pGEM-T Easy Vector (Promega). Cloned PCR products were sequenced using an ABI Prism 3730 XL DNA Analyzer (Applied Biosystems, Foster City, CA, USA). Sequences of the 5′- flanking regions were analyzed using the Basic Local Alignment Search Tool (BLAST) Assembled Genome database (http://blast.ncbi.nlm.nih.gov/BLAST.cgi) and the UCSC Genome Bioinformatics browser (http://www.genome.ucsc.edu) to identify transgene integration sites in the transgenic chickens’ genomes.

### Immunohistochemistry

The procedures of testis section and immunostaining were followed by our previous report^47^. Adult testes of WT chickens and busulfan-treated or untreated germline chimeric chickens were paraffin-embedded and sectioned (thickness, 10 μm). After deparaffinization, sections were washed three times with 1 × phosphate-buffered saline (PBS) and blocked with a blocking buffer (5% goat serum and 1% bovine serum albumin in PBS) for 1 h at room temperature. Sections were then incubated at 4 °C overnight with a rabbit anti-GFP primary antibody (Invitrogen, Carlsbad, CA, USA) (1:200 dilutions in blocking buffer). After washing three times with PBS, sections were incubated with fluorescence-conjugated secondary antibodies (Alexa Fluor 594 or 488, Invitrogen) for 1 h at room temperature. After washing three times with PBS, sections were mounted with Prolong Gold antifade reagent with DAPI and imaged using a confocal fluorescence microscope (Carl Zeiss Inc, Oberkocken, Germany).

### Statistical analyses

To analyze dose-dependent effects of busulfan on *MGSTII* PGCs and WT PGCs, a two-way ANOVA was used to determine statistical significance. Significant differences between busulfan-treated and busulfan non-treated groups were examined using a one-way ANOVA. *P* < 0.05 was considered indicative of statistical significance. ***p < 0.001, **p < 0.01, *p < 0.05.

### Ethics statement

All experimental procedures and care of chickens was approved by the Institute of Laboratory Animal Resources, Seoul National University, and all methods were carried out in accordance with ARRIVE (Animal Research: Reporting of In Vivo Experiments) guidelines and approved by the Institutional Animal Care and Use Committee (IACUC, SNU‐190,401‐1–1) of Seoul National University, Korea.

### Approval for animal experiments

All experimental procedures and care of chickens was approved by the Institute of Laboratory Animal Resources, Seoul National University (SNU‐190,401‐1–1), and all methods were carried out in accordance with guidelines and regulations of the Institutional Animal Care and Use Committee of Seoul National University (IACUC, SNU-200519–2), Korea. All procedures, including chicken maintenance, reproduction, and sample collection, were governed by standard operating protocols according to a standard management program at the University Animal Farm, Seoul National University and the Animal Genetic Engineering Laboratory at Seoul National University.

## Supplementary Information

Supplementary Information.
