# Supplementary material for: In vivo enrichment of busulfan-resistant germ cells for efficient production of transgenic avian models
Source: Sci Rep. 2021 Apr 28;11:9127. doi: 10.1038/s41598-021-88706-6 (PMC8080772; doi:10.1038/s41598-021-88706-6)
Supplement: Supplementary file 1 — Supplementary Information. [file 41598_2021_88706_MOESM1_ESM.pdf]

Title: *In vivo* enrichment of busulfan-resistant germ cells for efficient production of transgenic avian models

Young Min Kim[1*], Kyung Je Park[1*], Jin Se Park[1], Kyung Min Jung[1], and Jae Yong Han[1,2**]

[1]Department of Agricultural Biotechnology and Research Institute of Agriculture and Life Sciences, College of Agriculture and Life Sciences, Seoul National University, Seoul 08826, Korea

[2]Institute for Biomedical Sciences, Shinshu University, Minamiminowa, Nagano 399-4598, Japan

[**]Corresponding author: Jae Yong Han, Ph.D., Department of Agricultural Biotechnology, College of Agriculture and Life Sciences, Seoul National University, 1 Gwanak-ro, Gwanak-gu, Seoul 08826, Korea. Tel: +82-2-880-4810; Fax: +82-2-874-4811; E-mail: jaehan@snu.ac.kr

[*] These authors contributed equally to this article.

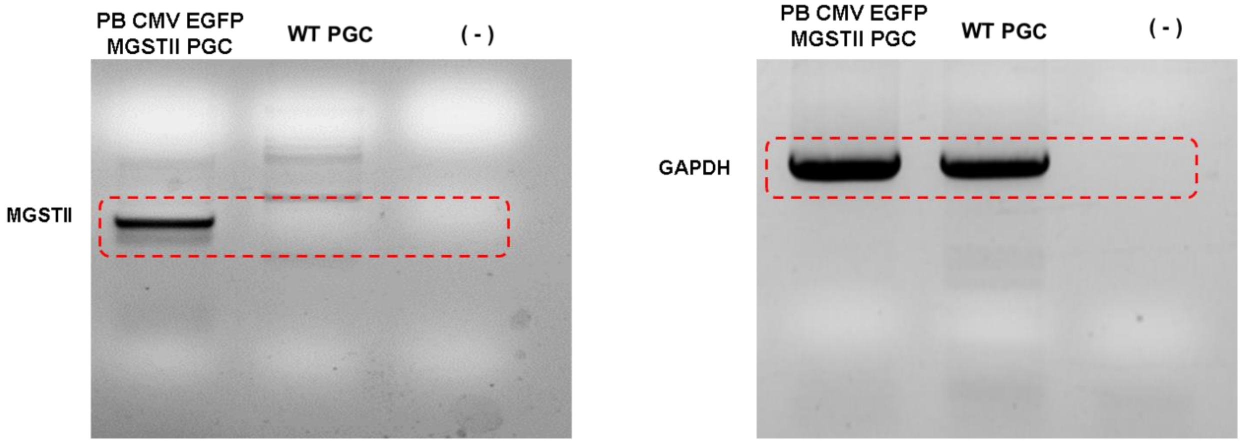

Supplementary Figure 1. Genomic DNA PCR analysis of *MGSTII-tg* PGCs using *MGSTII-* and *GAPDH*-specific primers. Wild-type (WT) PGCs treated with distilled water (-) were used as a control. The parts shown in Figure 1C are indicated by red dashed lines.

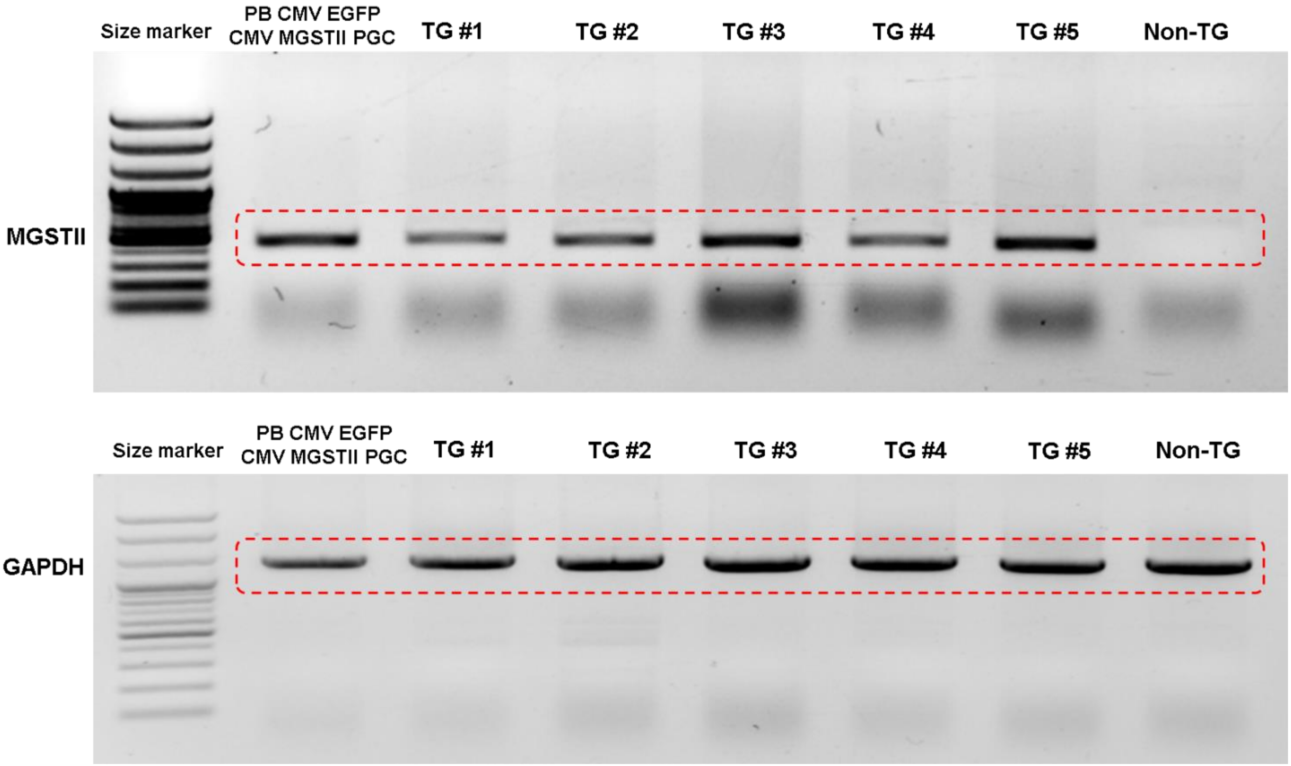

Supplementary Figure 2. Screening of *MGSTII-tg* chicks via genomic DNA PCR analysis. Genomic DNA PCR analysis of transgenic G_1 chicks using *MGSTII*- and *GAPDH*-specific primers. *MGSTII-tg* PGCs and non-transgenic genomic DNA samples were used as positive and negative controls, respectively. The parts shown in Figure 3B are indicated by red dashed lines.

Supplementary Table 1. Summary of the survivability and hatchability of KO recipients following transfer of PGCs with or without busulfan into embryonic blood vessels (HH 13–16)

Type of donor cells	With (+) or without (-) 1 μM busulfan treatment	No. of recipient embryos	No. of surviving embryos at embryonic day 6 (%)[†]	No. of hatched chicks (%)[‡]
**Control[#]**	-	10	10 (100)	10 (100)
**Busulfan only[*]**	+	8	4 (50.0)	4 (50.0)
***GFP-tg* PGCs**	-	8	7 (87.5)	5 (62.5)
	+	23	13 (56.5)	10 (43.5)
***MGSTII-tg* PGCs**	-	8	6 (100)	4 (50.0)
	+	22	19 (86.4)	13 (59.1)

[#]Control embryos grown and hatched without any treatment.

[*]Embryos injected with only 1 μM busulfan without any cells.

[†]The percentage of surviving embryos at 6 days after cell transplantation with/without busulfan. Surviving embryos were confirmed by the candling method using a bright light.

[‡]The percentage of hatched chickens after cell transplantation with/without busulfan.

Supplementary Table 2. Summary of experimental stock following injection of *MGSTII-tg* PGCs

Type of cells	No. of injected embryos	No. of hatched chicks	Wing tag ID (only male[‡])	Wing tag ID of sexually matured male germline chimeras
MGSTII-tg PGCs (male)	39	21	0396, 0398, 0399, 0411, 0412, 0415, 0416, 0418	0398, 0399, 0411, 0412, 0416

[‡]Only males were selected from Korean Ogye (KO) recipients as putative germline chimeras.
